# Multiple potassium channel tetramerization domain (KCTD) family members interact with Gβγ, with effects on cAMP signaling

**DOI:** 10.1016/j.jbc.2023.102924

**Published:** 2023-02-01

**Authors:** Douglas C. Sloan, Casey E. Cryan, Brian S. Muntean

**Affiliations:** Department of Pharmacology and Toxicology, Medical College of Georgia, Augusta University, Augusta, Georgia, USA

**Keywords:** G protein–coupled receptor, cell signaling, signal transduction, cAMP, adenylate cyclase, protein–rotein interaction, potassium channel tetramerization domain, KCTD, KCTD2, KCTD5, KCTD9, KCTD17, AC, adenylyl cyclase, AC5, AC type 5, BRET, bioluminescence resonance energy transfer, BTB, Bric-à-brac, Tramtrack, Broad complex, CAMPER, cAMP *E*ncoded *R*eporter, CC, coiled-coil, DIV, day *in vitro*, D2R, D2 dopamine receptor, FBS, fetal bovine serum, GIRK, G protein–gated inwardly rectifying potassium, GPCR, G protein–coupled receptor, HBSS, Hank’s balanced salt solution, HEK293, human embryonic kidney 293 cell line, IP, immunoprecipitation, KCTD, potassium channel tetramerization domain, Nluc, Nanoluciferase, PBST, PBS containing 0.1% Tween-20, PTX, pertussis toxin S1 subunit

## Abstract

G protein–coupled receptors (GPCRs) initiate an array of intracellular signaling programs by activating heterotrimeric G proteins (Gα and Gβγ subunits). Therefore, G protein modifiers are well positioned to shape GPCR pharmacology. A few members of the potassium channel tetramerization domain (KCTD) protein family have been found to adjust G protein signaling through interaction with Gβγ. However, comprehensive details on the KCTD interaction with Gβγ remain unresolved. Here, we report that nearly all the 25 KCTD proteins interact with Gβγ. In this study, we screened Gβγ interaction capacity across the entire KCTD family using two parallel approaches. In a live cell bioluminescence resonance energy transfer–based assay, we find that roughly half of KCTD proteins interact with Gβγ in an agonist-induced fashion, whereas all KCTD proteins except two were found to interact through coimmunoprecipitation. We observed that the interaction was dependent on an amino acid hot spot in the C terminus of KCTD2, KCTD5, and KCTD17. While KCTD2 and KCTD5 require both the *B*ric-à-brac, *T*ramtrack, *B*road complex domain and C-terminal regions for Gβγ interaction, we uncovered that the KCTD17 C terminus is sufficient for Gβγ interaction. Finally, we demonstrated the functional consequence of the KCTD–Gβγ interaction by examining sensitization of the adenylyl cyclase–cAMP pathway in live cells. We found that Gβγ-mediated sensitization of adenylyl cyclase 5 was blunted by KCTD. We conclude that the KCTD family broadly engages Gβγ to shape GPCR signal transmission.

G protein–coupled receptors (GPCRs) represent one of the most prominent mechanisms for cellular communication, controlling key physiological processes in almost every mammalian cell and tissue type (1). In a prototypic series of events, ligand-activated GPCRs induce the mobilization of heterotrimeric G proteins (Gα and obligatory Gβγ dimers) to engage effector molecules triggering downstream events (2, 3). Thus, modulation of active G protein lifetime critically dictates signaling magnitude and duration (4). In particular, Gβγ engages a host of effectors (5, 6, 7) on a spatiotemporal scale (8). This is exemplified in the case of Gβγ gating of K^+^ flux through G protein–gated inwardly rectifying potassium (GIRK) channels (9). The GPCR–GIRK signaling axis is finely tuned by proteins that either promote or reduce Gβγ availability (10), with one example pertaining to the binding of Gβγ by certain potassium channel tetramerization domain (KCTD) proteins (11, 12).

The KCTD family consists of 25 proteins that contain great diversity outside a structurally similar *B*ric-à-brac, *T*ramtrack, *B*road complex (BTB) domain, which organizes KCTD complex oligomerization (13, 14). The function of most KCTD proteins have remained relatively obscure, despite numerous ties to pathophysiological conditions (14, 15). In addition to regulating the GABA_B_–Gβγ–GIRK signaling axis by KCTD12 and KCTD16 (11, 12), there is a growing appreciation that numerous KCTDs serve as adapters that scaffold Cullin3 to mediate ubiquitination of target proteins (16, 17, 18, 19, 20). Several targets for KCTD-dependent ubiquitination have been described (19, 21, 22). Curiously, the best characterized substrate for ubiquitination is Gβγ (23), which is mediated through interaction with KCTD2 and KCTD5 (24). Indeed, loss of KCTD2 and KCTD5 (as well as KCTD17) leads to enhanced Gβγ-dependent second messenger signaling downstream through the cAMP pathway (25). Despite substantial functional information resulting from interactions between Gβγ with a few KCTDs (12), the molecular determinants underpinning such interaction are still unclear. Moreover, investigation into the remaining KCTD family toward Gβγ has yet to be defined.

In this study, we utilized two independent approaches to screen Gβγ interaction profiles across the entire KCTD family. Despite considerable diversity between KCTDs, we report that nearly every KCTD interacts with Gβγ though immunoprecipitation (IP), and about half of the KCTD family interact in an agonist-induced fashion in a bioluminescence resonance energy transfer (BRET)–based assay. We then utilized a subset of KCTD to further investigate rules of engagement for selectivity for interaction with Gβγ. We demonstrate how these principles enable KCTD2/5/9/17 to modulate Gβγ-dependent signal transmission in live cells. Finally, we report that various KCTDs modulate efficacy of cAMP signaling in primary striatal neurons.

## Results

### Detectable interaction between the majority of KCTD proteins with Gβγ

**Figure 1:**
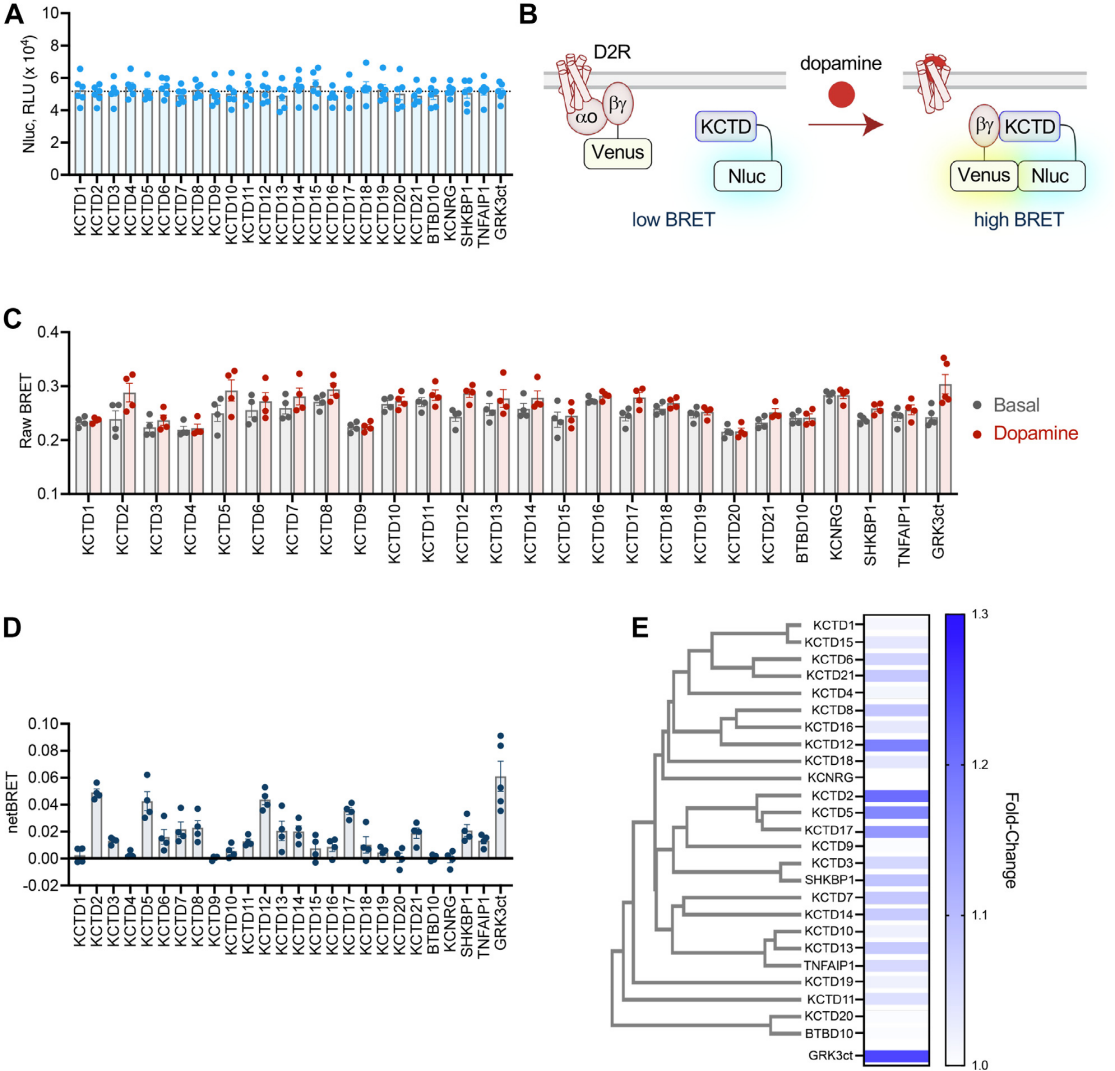
**Interaction profile of KCTD family with Gβγ in a live cell BRET-based assay.***A*, the intensity of bioluminescence following application of Nano-Glo reagent from transfection of HEK293 cells with KCTD-Nluc constructs in live cells. Intensity recorded as relative light units (RLUs). Data plotted as mean ± SEM from five biological replicates. *Dashed line* indicates average of all samples (5.169 × 10^4^). *B*, scheme of BRET assay to probe dopamine-induced interaction between C-terminal Nanoluciferase (Nluc)-tagged KCTD (KCTD-Nluc) and Gβγ–Venus in live cells. *C*, raw BRET ratio (535 nm Venus acceptor divided by 460 nm Nluc donor) from basal or 5 min after 100 μM dopamine incubation from cells transfected with D2R, GαoA, Venus 156-239-Gβ1, Venus 1-155-Gγ2, and Nluc-tagged constructs in a 0.5:1:1:1:1 ratio. Data plotted as mean ± SEM. n = 4 biological replicates (five replicates for Grk3ct) (≥2 technical replicates/sample) per sample. *D*, quantification of netBRET (basal BRET subtracted from dopamine BRET) following incubation in 100 μM dopamine for 5 min. Data plotted as mean ± SEM. n = 4 biological replicates (five replicates for Grk3ct) (≥2 technical replicates/sample) per sample. *E*, phylogenetic tree of human KCTD family aligned with BRET fold change plotted as a heatmap. GRK3ct value plotted as a reference. BRET, bioluminescence resonance energy transfer; KCTD, potassium channel tetramerization domain.

Combined functional and structural data strongly support KCTD engagement with Gβγ following GPCR activation (11, 12, 23, 26). Therefore, we began with an unbiased functional evaluation to determine which KCTD proteins could interact with Gβγ dimers. We devised a BRET assay to monitor GPCR agonist–induced interaction of KCTD with Gβγ in live cells. For the BRET donor, we fused Nanoluciferase (Nluc) to the C terminus of each full-length KCTD. Upon transfection in human embryonic kidney 293 (HEK293) cells and exposure to Nano-Glo substrate, each Nluc construct yielded similar bioluminescence intensity suggesting relatively equal KCTD expression level (Fig. 1*A*). We then utilized the well-characterized bimolecular fluorescence complementation Venus fluorophore split between Gβ1 (Venus 156-239-Gβ1) and Gγ2 (Venus 1-155-Gγ2) as the BRET acceptor strategy (27). In our assay, HEK293 cells were transiently transfected with KCTD-Nluc, Gβγ–Venus, D2 dopamine receptor (D2R), and GαoA (Fig. 1*B*). GPCR signal transmission was initiated by D2R activation with dopamine. We recorded the agonist-induced BRET response after 5 min and compared with basal readings (Fig. 1*C*). As a reference, we performed control experiments with Nluc fused to the C terminus of the GRK3 effector (GRK3ct), which exhibits nanomolar affinity for Gβγ (28) and readily reports agonist-induced association with Gβγ–Venus (29, 30). Approximately half of the KCTD family exhibited an increased BRET signal after dopamine application, suggesting interaction with Gβγ (Fig. 1*D*). Among these were several KCTDs previously identified in complex with Gβγ (KCTD2, KCTD5, and KCTD12). On the other hand, KCTD16, which has been demonstrated to bind Gβγ (11), generated only a mild BRET response in our experiments. However, KCTD16 elicited a relatively higher basal BRET, which could indicate interaction with Gβγ prior to receptor stimulation. In addition, we revealed numerous KCTDs that had not previously been known to engage Gβγ. To gain insight toward binding patterns, we generated a phylogenetic tree of the human KCTD family aligned with a heatmap of the BRET fold change (Fig. 1*E*). The analysis revealed consistency in Gβγ interaction between KCTD subgroups. One exception to this observation was the lack of BRET response with KCTD9, differing from the robust signal exhibited by similar group members (KCTD2, KCTD5, and KCTD17).

**Figure 2:**
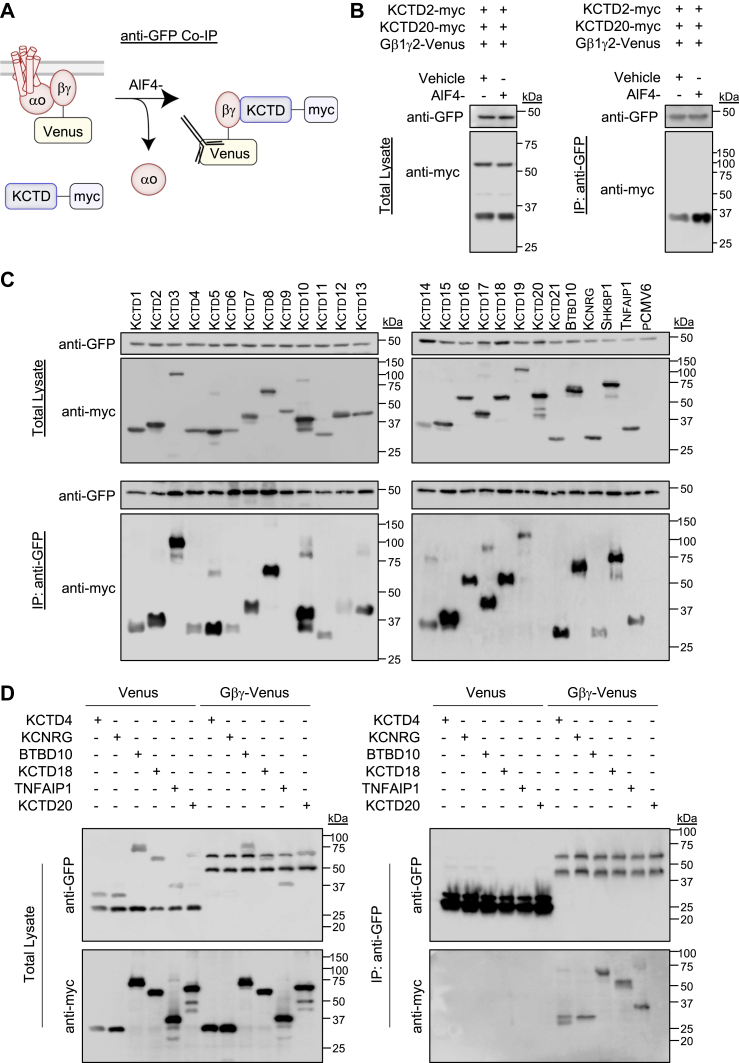
**Interaction profile of KCTD family with Gβγthrough immunoprecipitation (IP) in reconstituted cells.***A*, scheme of co-IP experiment from cells transfected with GαoA, Venus 156-239-Gβ1, Venus 1-155-Gγ2, and KCTD-myc constructs in a 1:1:1:1 ratio. AlF4- (30 μM) was utilized in lysis buffer to dissociate heterotrimeric G proteins during pulldown with anti-GFP antibody. *B*, IP of Gβγ–Venus complexes from HEK293 cells with anti-GFP antibody followed by probing for C-terminal myc-tagged KCTD2 and KCTD20 with an anti-myc antibody. AlF4- (30 μM) increased co-IP of KCTD2 (∼32 kDa predicted weight) but did not enable detection KCTD20 (∼50 kDa predicted weight). Representative blot from three independent experiments. *C*, IP of Gβγ–Venus complexes from HEK293 cells with anti-GFP antibody followed by probing for detection of C-terminal myc-tagged KCTD with an anti-myc antibody. Experiments were performed in the presence of AlF4- (30 μM) in the lysis buffer. Representative blot from three independent experiments. *D*, IP of either Venus (∼27 kDa predicted weight) or Gβγ–Venus (∼47 kDa predicted weight) complexes from HEK293 cells with anti-GFP antibody followed by probing for detection of C-terminal myc-tagged KCTD with an anti-myc antibody. Experiments were performed in the presence of AlF4- (30 μM) in the lysis buffer. Representative blot from three independent experiments. HEK293, human embryonic kidney 293 cell line; KCTD, potassium channel tetramerization domain.

Given that KCTD16 yielded a small netBRET and relatively higher basal BRET, we wanted to ensure that our approach did not limit detection of KCTD–Gβγ interactions. Therefore, we next utilized IP to examine the capacity of KCTD to interact with Gβγ. In this experiment, we fused a myc tag to the carboxy terminus of the KCTD ORF for transfection into HEK293 cells in tandem with GαoA and Gβγ–Venus. Cells were then lysed followed by pulldown with a GFP antibody. We first tested whether promoting heterotrimeric G protein dissociation would enhance detection of in the KCTD–Gβγ interactions and therefore performed lysis/IP in the presence or the absence of AlF4- (Fig. 2*A*). For this purpose, cells were cotransfected with KCTD2 (highest netBRET) and KCTD20 (no netBRET). Probing the total lysate with an anti-myc antibody revealed similar KCTD expression level corresponding to the estimated molecular weight (Fig. 2*B*). Treatment with AlF4- increased the KCTD2 band intensity in the IP samples; however, KCTD20 was not detected in the IP regardless the treatment. The result fortifies the BRET observation for KCTD2/KCTD20, suggests AlF4- may enhance the detection window for KCTD–Gβγ interactions through co-IP, and demonstrates AlF4- treatment will not induce detection of false positives (at least in the case of KCTD20).

Therefore, we applied the IP strategy with AlF4- to the entire KCTD family. Probing total lysates with a myc-tag antibody demonstrated expression of each KCTD near the predicted molecular weight (Fig. 2*C*). Stunningly, the IP samples revealed interaction of Gβγ with all but two KCTD proteins (KCTD9 and KCTD20). Although not quantitative, the IP results trend toward categories of stronger (KCTD2, 3, 5, 7, 8, 10, 15, 16, 17, 18, BTBD10, SHKBP1) and weaker (KCTD1, 4, 6, 11, 12, 19, KCNRG, TNFAIP1) interactions with Gβγ. Therefore, we examined the possibility of nonspecific interactions by repeating the Gβγ–Venus IP in parallel with Venus-transfected cells. For this experiment, we utilized three weak binders (KCTD4, KCNRG, and TNFAIP1), two strong binders (BTBD10 and KCTD18), and one nonbinder (KCTD20). While protein levels were similar in total lysates across the board, no KCTDs were detected in Venus IP, whereas all but KCTD20 were detected in the Gβγ–Venus IP (Fig. 2*D*).

The results collectively show that while at least half of the KCTD family were observed to engage Gβγ in an agonist-induced fashion in live cells, nearly all KCTDs have the capacity for Gβγ interaction through IP. While KCTD interactions with Gβγ may vary in strength, control experiments suggest they do not appear to be false positives.

### KCTD interaction with Gβγ requires BTB and C-terminal domains

**Figure 3:**
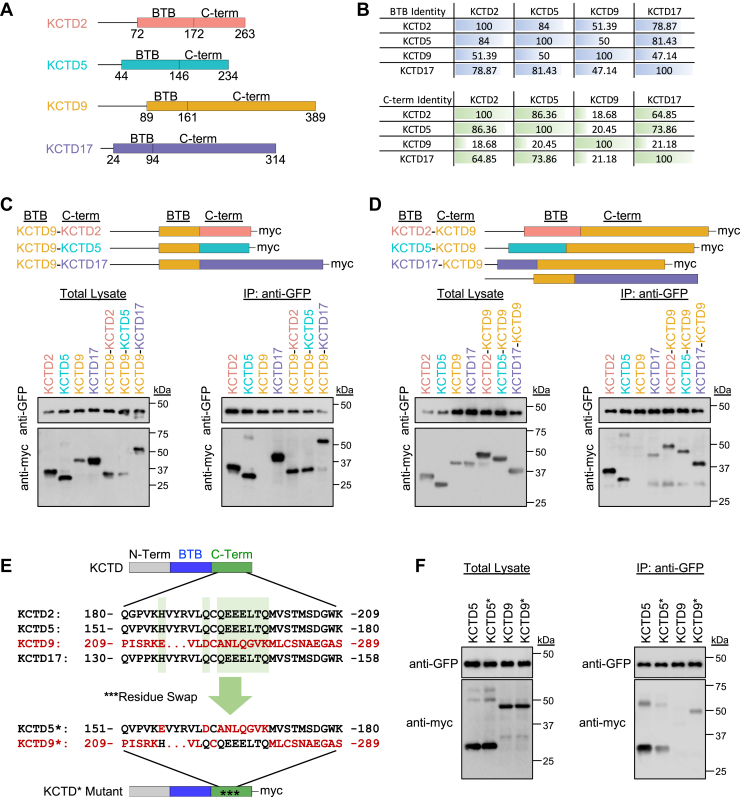
**KCTD requires BTB and C-terminal domains for interaction with Gβγ.***A*, scheme of KCTD architecture highlighting BTB domain relative to N and C terminus. *B*, BTB- and C-terminal amino acid identity shared between human KCTD 2, 5, 9, and 17. *C*, immunoprecipitation (IP) of Gβγ–Venus complexes from HEK293 cells with anti-GFP antibody followed by probing for detection of C-terminal myc-tagged KCTD9 chimeras with C terminus from KCTD 2, 5, or 17 with an anti-myc antibody. Experiments were performed in the presence of AlF4- (30 μM) in the lysis buffer. Representative blot from three independent experiments. *D*, IP of Gβγ–Venus complexes from HEK293 cells with anti-GFP antibody followed by probing for detection C-terminal myc-tagged KCTD9 chimeras with BTB domain from KCTD 2, 5, or 17 with an anti-myc antibody. Experiments were performed in the presence of AlF4- (30 μM) in the lysis buffer. Representative blot from three independent experiments. *E*, identification of charged/polar residues in C terminus of KCTD 2, 5, and 17 that are not conserved in KCTD9. Diagram depicts mutated KCTD5∗ and KCTD9∗ where highlighted residues are swapped. *F*, IP of Gβγ–Venus complexes from HEK293 cells with anti-GFP antibody followed by probing for detection of C-terminal myc-tagged KCTD5∗ and KCTD9∗ proteins. Experiments were performed in the presence of AlF4- (30 μM) in the lysis buffer. Representative blot from three independent experiments. BTB, *B*ric-à-brac, *T*ramtrack, *B*road complex; HEK293, human embryonic kidney 293 cell line; KCTD, potassium channel tetramerization domain.

We next sought to understand molecular determinants that enable broad KCTD interaction with Gβγ. Of the noninteractors, KCTD20 shares isolated similarity with BTBD10, whereas KCTD9 belongs to a subfamily that includes several members (KCTD2, KCTD5, and KCTD17) as well as conservation between other clades (Figs. 1*C* and S1). Therefore, we reasoned that differences in amino acids between KCTD9 and its subfamily could reveal distinct signatures that enable interaction with Gβγ. Given the paucity of information on KCTD9 interaction profiles, we started by reducing the KCTD architecture to its simplest shared domains: (i) varying length N terminus, (ii) BTB domain, (iii) remaining C terminus (Fig. 3*A*). Comparison of BTB domains revealed ∼50% identity between KCTD9 with KCTD2, KCTD5, or KCTD17, whereas KCTD2, KCTD5, and KCTD17 exhibited greater than 75% identity between each other (Fig. 3*B*). Similarly, KCTD2, KCTD5, and KCTD17 exhibit high identity between their C termini (>60%), whereas KCTD9 exhibits low C-terminal identity (∼20%) with KCTD2, KCTD5, and KCTD17 (Fig. 3*B*). Therefore, we hypothesized that selectivity for Gβγ interaction may be conferred by the C terminus. We made chimeras of myc-tagged KCTD9 by replacing its C terminus with that of KCTD2, KCTD5, or KCTD17. We then repeated IP experiments with Gβγ–Venus. Indeed, we observed that each KCTD9 chimera was able to interact with Gβγ (Fig. 3*C*). We next tested if the C terminus of KCTD9 would impede interaction with Gβγ. Thus, we made chimeras of myc-tagged KCTD2, KCTD5, and KCTD17 by replacing their C terminus with that of KCTD9. Interestingly, we found that these chimeras retained the ability to interact with Gβγ in our IP experiment (Fig. 3*D*), suggesting interplay between elements within the BTB and C-terminal regions of KCTD2, KCTD5, and KCTD17. We next performed a bioinformatics analysis to understand which components enable Gβγ interaction. We identified a region of charged and polar amino acids in the C terminus of KCTD2, KCTD5, and KCTD17 that were not conserved in KCTD9 (Fig. 3*E*). We made a myc-tagged KCTD5 mutant where we swapped these residues with the ones from KCTD9. Likewise, we replaced these residues in myc-tagged KCTD9 with their counterpart from KCTD5 and then performed the IP experiments. We found that while the KCTD5 mutant was still able to interact with Gβγ (although seemingly lesser than wildtype), the presence of the charged/polar residues on KCTD9 began to enable binding capacity for Gβγ as well (Fig. 3*F*). These experiments collectively suggest that the KCTD2/5/9/17 clade require key residues within both the BTB domain and C terminus in order to interact with Gβγ.

### KCTD17 C terminus sufficient for Gβγ interaction

**Figure 4:**
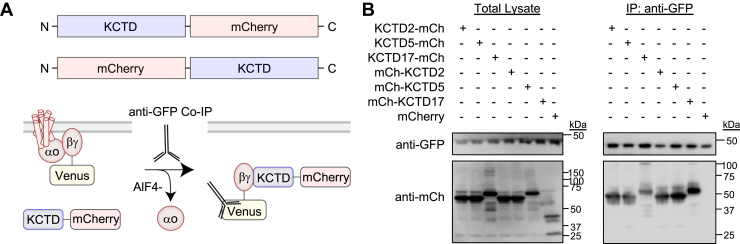
**Neither N- or C-terminal placement of epitope tag interferes with KCTD–Gβγ interaction.***A*, scheme of N- and C-terminal placement of mCherry on full-length KCTD for co-IP experiment with Gβγ. *B*, IP of Gβγ–Venus complexes from HEK293 cells with anti-GFP antibody followed by probing for detection of mCherry-tagged KCTD with an anti-mCherry antibody. pmCherry-N1 transfected cells were utilized as a control. Experiments were performed in the presence of AlF4- (30 μM) in the lysis buffer. Representative blot from three independent experiments. HEK293, human embryonic kidney 293 cell line; IP, immunoprecipitation; KCTD, potassium channel tetramerization domain.

**Figure 5:**
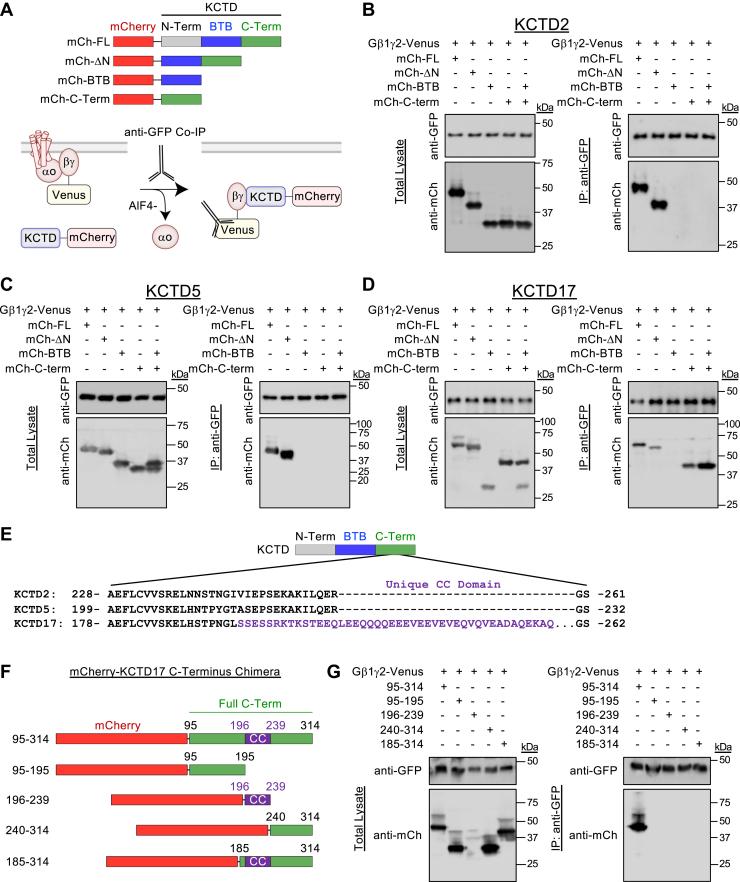
**KCTD17 C terminus is sufficient for Gβγ interaction.***A*, scheme of N-terminal mCherry constructs utilized (FL = full length, ΔN= deletion of N terminus, BTB = BTB domain only, C-Term = C terminus only). *B*, immunoprecipitation (IP) of Gβγ–Venus complexes from HEK293 cells with anti-GFP antibody followed by probing for mCherry-KCTD2 proteins with an anti-mCherry antibody. Experiments were performed in the presence of AlF4- (30 μM) in the lysis buffer. Representative blot from three independent experiments. *C*, IP of Gβγ–Venus complexes from HEK293 cells with anti-GFP antibody followed by probing for mCherry-KCTD5 proteins with an anti-mCherry antibody. Experiments were performed in the presence of AlF4- (30 μM) in the lysis buffer. Representative blot from three independent experiments. *D*, IP of Gβγ–Venus complexes from HEK293 cells with anti-GFP antibody followed by probing for mCherry-KCTD17 proteins with an anti-mCherry antibody. Experiments were performed in the presence of AlF4- (30 μM) in the lysis buffer. Representative blot from three independent experiments. *E*, alignment of C-terminal animo acid residues in KCTD2, 5, 17. *Purple region* highlights coiled-coil domain unique to KCTD17. *F*, scheme of constructs utilized that contain an N-terminal mCherry fused to the indicated C-terminal animo acids of KCTD17. *G*, IP of Gβγ–Venus complexes from HEK293 cells with anti-GFP antibody followed by probing for mCherry-KCTD17 C-terminal proteins with an anti-mCherry antibody. Experiments were performed in the presence of AlF4- (30 μM) in the lysis buffer. Representative blot from three independent experiments. HEK293, human embryonic kidney 293 cell line; KCTD, potassium channel tetramerization domain.

We next determined if tag placement on KCTD influenced interaction with Gβγ. For this purpose, we fused mCherry to either the N terminus or C terminus of KCTD2, KCTD5, and KCTD17 (Fig. 4*A*). Placement of mCherry at either position yielded equivalent expression in the total lysate as well as interaction profile with Gβγ through IP (Fig. 4*B*). Next, we asked if the BTB domain or C terminus alone was sufficient for interaction with Gβγ–Venus in our IP experiments. We mapped these regions by generating N-terminal mCherry tags on KCTD2, KCTD5, and KCTD17 (Fig. 5*A*). We utilized the full-length construct as a control (mCh-FL), deleted the N terminus (mCh-ΔN), expressed only the BTB domain (mCh-BTB), or expressed only the C terminus (mCh-C-term). Starting with KCTD2, we found that the N terminus was dispensable for interaction with Gβγ (Fig. 5*B*). However, the BTB domain and C terminus were not sufficient for Gβγ interaction. Interestingly, coexpression of BTB domain and C terminus from separate vectors was also unable to provide interaction with Gβγ. We found that KCTD5 elicited the same interaction profile (Fig. 5*C*), likely owing to high degree of similarity with KCTD2. We next mapped KCTD17, which showed that while the N terminus was not required for interaction with Gβγ, the C terminus was sufficient (Fig. 5*D*). Intriguingly, the C terminus of KCTD17 contains a coiled-coil (CC) domain that is not conserved with other KCTDs (Fig. 5*E*). Therefore, we generated N-terminal mCherry fusion constructs mapping regions of the KCTD17 C terminus to investigate involvement of this region (Fig. 5*F*). Unfortunately, the mCherry-CC domain did not express well following transfection compared with other regions of the KCTD17 C terminus (Fig. 5*G*). Moreover, only the full-length KCTD17 C terminus (95–314) was able to interact with Gβγ (Fig. 5*G*). Thus, these interaction studies demonstrate that the KCTD N terminus is not required for interaction with Gβγ; however, the C terminus of KCTD17 is sufficient for binding in the absence of a BTB domain.

### KCTD blocks Gβγ sensitization of AC type 5

**Figure 6:**
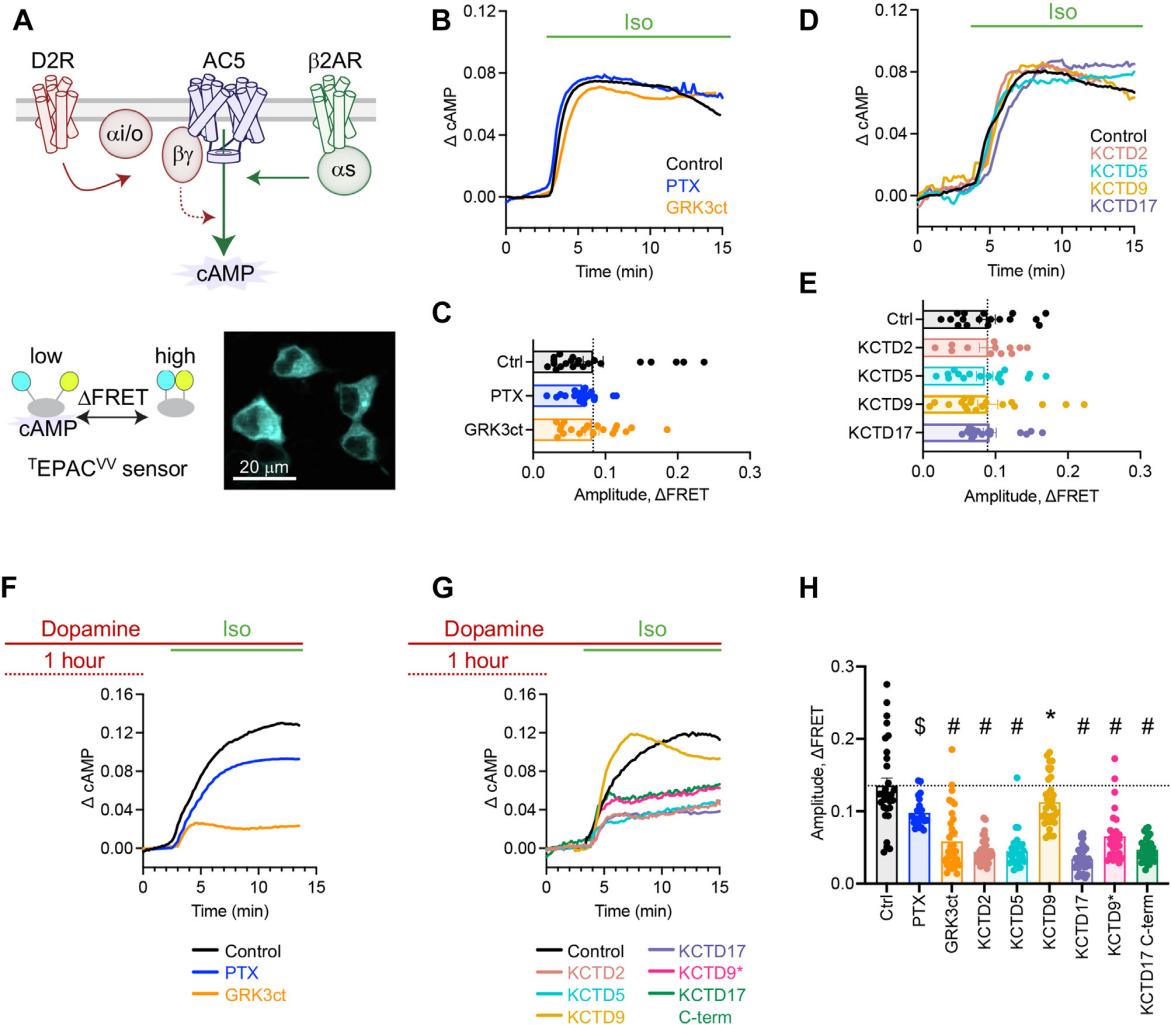
**KCTD overexpression attenuates cAMP sensitization in reconstituted cells.***A*, scheme of G protein inputs shaping signaling downstream to cAMP, which was recorded through FRET imaging of the ^T^Epac^VV^ sensor in HEK293 cells. Cells were cotransfected with D2R, AC5, and ^T^Epac^VV^, and PTX-S1/GRK3ct/KCTD/empty vector in a 1:1:1:2 ratio. *B*, representative cAMP responses to bath application of 1 μM isoproterenol from individual cells (n = 22 cells [Ctrl], 22 cells [PTX], 19 cells [GRK3ct]) collected from four independent experiments. *C*, maximum cAMP response amplitude. n = 22 cells (Ctrl), 22 cells (PTX), 19 cells (GRK3ct); one-way ANOVA; *p* > 0.05. Data collected from four independent experiments. *D*, representative cAMP responses to bath application of 1 μM isoproterenol from individual cells (n = 17 cells [Ctrl], 12 cells [KCTD2], 16 cells [KCTD5], 18 cells [KCTD9], 16 cells [KCTD17]; one-way ANOVA; *p* > 0.05). Data collected from three independent experiments. *E*, maximum cAMP response amplitude. n = 17 cells (Ctrl), 12 cells (KCTD2), 16 cells (KCTD5), 18 cells (KCTD9), 16 cells (KCTD17); one-way ANOVA; *p* > 0.05. Data collected from three independent experiments. *F*, representative cAMP responses to bath application of 1 μM isoproterenol following 1 h incubation with 100 μM dopamine from individual cells (n = 31 cells [Ctrl], 22 cells [PTX], 36 cells [GRK3ct]). Data collected from three independent experiments. *G*, representative cAMP responses to bath application of 1 μM isoproterenol following 1 h incubation with 100 μM dopamine from individual cells (n = 31 cells [Ctrl], 34 cells [KCTD2], 29 cells [KCTD5], 37 cells [KCTD9], 35 cells [KCTD17], 28 cells [KCTD9∗], 35 cells [KCTD17 C-term]). Data collected from three to four independent experiments. *H*, maximum cAMP response amplitude. n = 31 cells (Ctrl), 34 cells (KCTD2), 29 cells (KCTD5), 37 cells (KCTD9), 35 cells (KCTD17), 28 cells (KCTD9∗), 35 cells (KCTD17 C-term); one-way ANOVA; ∗*p* < 0.05, $*p* < 0.001, #*p* < 0.0001. Data collected from three to four independent experiments. AC5, adenylyl cyclase type 5; D2R, D2 dopamine receptor; HEK293, human embryonic kidney 293 cell line; KCTD, potassium channel tetramerization domain; PTX, pertussis toxin S1 subunit.

We next wanted to investigate downstream consequences of KCTD interaction with Gβγ in the context of intracellular GPCR signaling. For this purpose, we examined real-time cAMP dynamics by imaging the ^T^Epac^VV^ FRET-based biosensor in live HEK293 cells (31, 32). While numerous inputs shape adenylyl cyclase (AC)–mediated cAMP production, sensitization of AC type 5 (AC5) following prolonged Gi/o signaling involves Gβγ (33, 34). Therefore, we first optimized conditions to robustly interrogate Gβγ influence on cAMP. For each experiment, we overexpressed D2R, AC5, and ^T^Epac^VV^ (Fig. 6*A*). Stimulation of the endogenous Gαs-coupled β_2_-adrenergic receptor with 1 μM isoproterenol generated a robust increase in cAMP (Fig. 6*B*). The response was similar in cells with overexpression of a Gi/o inhibitor (pertussis toxin S1 subunit; PTX) or a Gβγ scavenger (GRK3ct) (Fig. 6*C*). In these transfection conditions, KCTD (KCTD2, KCTD5, or KCTD17) overexpression also did not alter isoproterenol-induced responses compared with control cells (Fig. 6, *D* and *E*). We next induced sensitization of AC5 by stimulating D2R with 100 μM dopamine for 1 h, which resulted in an enhanced cAMP response following subsequent application of 1 μM isoproterenol (Fig. 6*F*). PTX blocked the sensitized response resulting in similar amplitude to non-dopamine treated cells. GRK3ct blocked sensitization in addition to generating a significantly smaller amplitude than PTX-treated cells, likely because of ongoing AC5 inhibition by D2R→Gαi signaling. Overall, the results suggest a cAMP readout dependent on Gβγ, and we therefore next assessed the impact of KCTD overexpression. In agreement with our interaction studies, we found that cAMP sensitization was blunted by KCTD2, KCTD5, and KCTD17 (Fig. 6*G*). Curiously, KCTD9 slightly inhibited cAMP sensitization, suggesting a potential non-Gβγ effect (Fig. 6*H*). We also found that the KCTD9 mutant containing charged/polar residues from KCTD2, KCTD5, and KCTD17 decreased cAMP amplitude (Fig. 6*H*). Finally, the KCTD17 C terminus blunted cAMP response consistent with data obtained from the GRK3ct scavenger (Fig. 6*H*). Overall, these experiments support a model that KCTD interaction with Gβγ sequesters AC5-mediated sensitization of cAMP.

### KCTD modulates dopamine efficacy in D1R+ striatal neurons

**Figure 7:**
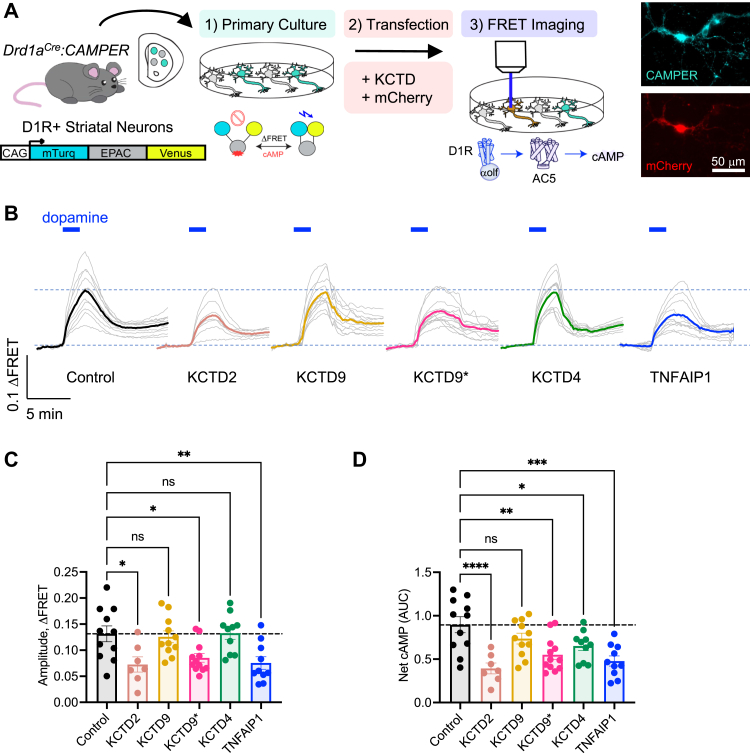
**KCTD overexpression reduces dopamine efficacy in D1R+ primary striatal neurons.***A*, scheme of experimental design. Primary striatal neurons were cultured from P0 pups (*Drd1aCre-CAMPER*^*+/+*^ crossed with *CAMPER*^*+/+*^). At DIV5, the cultures were transfected with KCTD-myc and pmCherry-N1 (1 μg each) for FRET imaging from DIV14 to 18. *B*, cAMP response from application of 100 μM dopamine. *Dotted horizontal lines* indicate baseline and peak response of control group as a reference. *Light traces* represent each individual neuronal responses, and *dark trace* is average of all neurons in the group: n = 11 (control), 7 (KCTD2), 11 (KCTD9), 12 (KCTD9∗), 10 (KCTD4), and 10 (TNFAIP1). Data collected from three independent batches of primary cultures. *C*, maximum cAMP response amplitude. n = 11 (control), 7 (KCTD2), 11 (KCTD9), 12 (KCTD9∗), 10 (KCTD4), 10 (TNFAIP1); one-way ANOVA; ∗*p* < 0.05, ∗∗*p* < 0.01. Data collected from three independent batches of primary cultures. *D*, net cAMP flux calculated as area under the curve of the response. n = 11 (control), 7 (KCTD2), 11 (KCTD9), 12 (KCTD9∗), 10 (KCTD4), 10 (TNFAIP1); one-way ANOVA; ∗*p* < 0.05, ∗∗*p* < 0.01, ∗∗∗*p* < 0.001, ∗∗∗∗*p* < 0.0001. Data collected from three independent batches of primary cultures. D1R, dopamine 1 receptor; DIV5, day *in vitro* 5; KCTD, potassium channel tetramerization domain.

As data in the reconstituted system support a role for KCTD overexpression to uncouple Gβγ from AC5, we next wanted to measure such impact on endogenous signaling. For this purpose, we utilized primary striatal neurons because AC5 is the major cyclase accounting for ∼80% of cAMP synthesis in these cells (35) where enzymatic activity is tuned by Gβγ (36, 37). We utilized the *cAMP E*ncoded *R*eporter (*CAMPER*) mouse line, which conditionally expresses the ^T^Epac^VV^ sensor. Because subpopulations of striatal neurons differentially express dopamine receptor subtypes, we isolated the stimulatory D1R→AC5→cAMP pathway by crossing homozygous *CAMPER (CAMPER*^*+/+*^*)* with a D1R+ Cre line (*Drd1aCre*) (32). Primary striatal neurons were then cultured from P0 pups (*Drd1aCre-CAMPER*^*+/+*^ and *CAMPER*^*+/+*^ breeding pairs), transfected with KCTD (and mCherry for visualization) at day *in vitro* (DIV) 5, and performed FRET imaging between DIV14 and DIV18 (Fig. 7*A*). KCTD2 was investigated because of its prominence as a strong interactor with Gβγ. Likewise, we selected KCTD9 (noninteractor) and the KCTD9 mutant (KCTD9∗) containing charged/polar residues that confer interaction with Gβγ. Finally, we wanted to explore KCTD that were observed to weakly interact with Gβγ and utilized the phylogenetically distinct KCTD4 and TNFAIP1 for such experiments. Bath application of a saturating dose of dopamine (100 μM) generated a robust increase in cAMP in each group of neurons (Fig. 7*B*). However, the response amplitude was significantly reduced by exogenous expression of KCTD2, KCTD9∗, and TNFAIP1 compared with control (Fig. 7*C*). Response amplitude was not altered by KCTD9 or KCTD4. To account for converge of multiple parameters contributing to signaling characteristics, we calculated net cAMP flux (area under the curve) (Fig. 7*D*). Here, we observed that the wave of cAMP response was significantly reduced in each condition except KCTD9. These data collectively support a role for distinct KCTDs, even those with relatively mild Gβγ interaction profiles, in regulating cAMP signaling in a physiological setting.

## Discussion

Signaling through GPCRs is one of the most prominent mechanisms for cellular communication, which relies on signal transduction mediated by Gα and Gβγ engagement with effector proteins. Therefore, molecules that interact with G proteins are well positioned to adjust downstream second messenger signaling profiles. Recent studies have identified examples of Gβγ subunits interacting with certain KCTD (2, 5, 12, 16) (11, 23, 24), which represent a family of proteins hallmarked by the presence of a BTB domain thought to endow facilitation of protein–protein interactions. However, it remains unclear the breadth of the KCTD family capacity for interaction with Gβγ, much less the molecular determinants guiding such interactions. In this study, we screened the entire family of KCTD proteins for their ability to interact with Gβγ. Utilizing a BRET-based live cell assay, we found that approximately half of the KCTD interact with Gβγ in an agonist-induced fashion. When examined through IP, we report nearly every KCTD capable of such interaction with exception pertaining to KCTD9 and KCTD20. Intriguingly, KCTD9’s homologous subfamily (KCTD2, KCTD5, and KCTD17) exhibited the strongest interaction profile in the BRET assay and IP. Therefore, utilizing these KCTDs as a case study, we extracted general rules and unique amino acid compositions required for interaction with Gβγ. We then applied insight in KCTD–Gβγ interaction to probe downstream consequences on GPCR signaling in live cells and primary neurons.

### Molecular determinants of KCTD–Gβγ interaction

A major finding in this study was elucidating that nearly every KCTD interacts with Gβγ, although there appears to be a spectrum of stronger and weaker binders. Nonetheless, the observation is particularly intriguing given the lack of similarity between KCTD aside from structurally conserved BTB domain, which ranges from 10 to 40% identity across the KCTD family. The BTB domain on KCTD shares homology with the T1 domain on K_V_-type potassium channels (13). Both domains are thought to facilitate protein–protein interactions. In the case of Kv, T1 enables channel formation *via* tetramerization (38). Similarly, BTB interactions between KCTD result in oligomerization observed in crystal structures: KCTD1 (open and closed pentamer) (39), KCTD5 (closed pentamer) (40), KCTD9 (closed pentamer) (39), KCTD10 (symmetric tetramer) (41), KCTD12 (closed pentamer) (12), KCTD13 (symmetric tetramer) (41), KCTD16 (open pentamer and closed hexamer) (13, 26, 41), and KCTD17 (closed pentamer) (41). It should be noted that at least one KCTD has been observed to exist as a monomer (SHKBP1) (41).

Our data support a general model for interaction with Gβγ where the BTB domain provides structural foundation of KCTD oligomers, which orients the C terminus to confer selectivity toward interaction with Gβγ. This is supported by several lines of evidence. In the case of KCTD2 and KCTD5, neither the BTB domain nor C terminus were sufficient for interaction with Gβγ. Rather, expression of both components *in cis* was necessary, and no interaction with Gβγ was observed when the BTB and C terminus were expressed *in trans* as separate proteins. Components in both the BTB domain and C terminus rendered KCTD9 unable to interact with Gβγ. We were thus able to unlock KCTD9 interaction with Gβγ by replacing either the BTB domain or C terminus with that of KCTD2, KCTD5, or KCTD17; further suggesting motifs in both regions of KCTD are required. To fortify this conclusion, we identified a stretch of surface-exposed charged/polar amino acids on the C terminus of the KCTD5 structure (40) that are conserved between KCTD2 and KCTD17 but not with KCTD9 (or any other KCTD). Mutating this region enabled KCTD9 interaction with Gβγ and appeared to decrease the affinity of KCTD5 for Gβγ, although the latter observation was not quantitative. Further evidence that BTB domains do not bind Gβγ were also described in the case of an H1 domain that is exclusive to the C terminus of KCTD8, KCTD12, and KCTD16 (42). The H1 domain was found to be both necessary and sufficient for direct binding to Gβγ (12). Finally, we made a similar observation in the case of KCTD17 where the C terminus alone was sufficient for interaction with Gβγ. The C-terminal domain on KCTD17 contains a CC domain that is not conserved with KCTD2 and KCTD5 (or KCTD9), which may endow interaction for Gβγ. Attempts to pulldown Gβγ with the KCTD17 CC were not successful as our fusion construct did not express well. The C termini of KCTD have further been implicated in interaction with additional target proteins: KCTD5-GRASP55 (40), KCTD5-C5aR1 (43), and KCTD17-trichoplein (19). On the other hand, the BTB domain has been implicated in interaction with Cullin3 (17, 44, 45); however, this is thought to be due to the presence of a BTB domain on Cullin3 as well (46). Intriguingly, Cullin3 interaction with KCTD is not a universal feature (20). Regardless, we cannot rule out key roles for BTB domain participation in intermolecular interactions. Indeed, perhaps the best characterized example pertains to the subfamily of KCTD8, KCTD12, and KCTD16. Crystal structures have demonstrated that GABA_B_ interacts directly with the BTB domain of KCTD12 and KCTD16 (12, 26).

Curiously, when we studied the interaction in live cells with a BRET-based approach, we found that only approximately half of the KCTD family exhibited robust interaction with Gβγ. This could be for several reasons. Our BRET experiments utilized the dopamine D2 receptor, and GPCRs may exert intrinsic bias toward individual KCTDs, which could be defined at the receptor level. As mentioned, KCTD12 and KCTD16 are tethered to the membrane via GABA_B_, thus promoting access to Gβγ dimers to modulate effector signaling (42). Therefore, KCTD8, KCTD12, and KCTD16 may favor interaction with Gβγ following signal transmission through GABA_B_ rather than D2R. However, we observed robust BRET responses with both KCTD8 and KCTD12, which may suggest some promiscuity between signal regulation by specific GPCRs and KCTD members. Indeed, KCTD12 has also been shown to rapidly desensitize potassium currents mediated through the adenosine 1 receptor in reconstituted Chinese hamster ovary cells (11). It is also possible that some KCTD may interact with Gβγ prior to GPCR stimulation. Indeed KCTD16 exhibited a high basal BRET reading with subsequently low netBRET and yet was robustly pulled down with Gβγ in our IP experiments. Other KCTDs fit this pattern as well (*e.g.*, KCTD10, KCTD11, KCTD18). An additional consideration may be the affinity of each KCTD for Gβγ, of which widespread information is not yet available. As a benchmark however, the isothermal titration calorimetry *K*_*d*_ measurement for the H1 domain of KCTD12 binding to Gβ1γ2 has been reported and is roughly 185 nM (12). Therefore, KCTD interplay with receptors or possibly even scaffolding complexes as well as affinity for Gβγ are all factors likely to shape interaction profiles. Finally, there are technical aspects regarding the assay that merit discussion. Experiments were performed with the BRET donor on the C terminus of KCTD and with the BRET acceptor on Gβ1γ2. Placing the BRET donor on the KCTD N terminus could potentially influence the readout. Moreover, switching the BRET players such that the Nluc donor was configured on Gβ1γ2 and Venus acceptor on KCTD may further adjust the response window.

### Signaling perspective

Our study further illustrates the impact that KCTD interaction with Gβγ exerts on modulating GPCR signal transmission. Prolonged activation of Gi/o signaling sensitizes AC5-mediated cAMP production in a process dependent on Gβγ subunits (47). Sensitized AC responses have been observed with addition of recombinant Gβγ (33, 48) or increasing available Gβγ by removal of regulator of G protein signaling proteins (49). In addition, the sensitization phenomenon is attenuated by blocking Gi/o transduction (*e.g.*, pertussis toxin) (50) or scavenging Gβγ dimers (*e.g.*, GRK3ct) (51). Here, we observed overexpression of KCTD (2, 5, or 17) that interact with Gβγ blunted AC5 sensitization. The same experiment with KCTD9, which did not interact with Gβγ, mildly reduced sensitization. Yet experiments with the KCTD9 mutant containing charged/polar residues that enable Gβγ interaction blocked cAMP sensitization similar to other KCTDs (2, 5, or 17). Thus, KCTD9 may also exhibit non-Gβγ effects contributing to cAMP signaling that are beyond the scope of this work. Nonetheless, the data reinforce the notion that KCTD interaction with Gβγ modulates cellular signaling and likely a couple of mechanisms contribute to the specific effect on cAMP. On one hand, scavenging Gβγ enables KCTD to block sensitization similar to observations made with GRK3ct. However at the same time, KCTD2 and KCTD5 are also adapters for Cullin3 that target ubiquitination and degradation of Gβγ, thus further reducing availability for sensitized cAMP responses (24). In previous work, we examined the opposite hypothesis. We demonstrated that GPCR stimulation of cAMP generation was significantly augmented during loss of KCTD (2, 5, or 17) in primary striatal neurons, an effect attributed to increased level of Gβγ (25). Here, we found the corollary effect of KCTD overexpression in primary striatal neurons: KCTDs that interact with Gβγ significantly reduced stimulatory dopamine receptor signaling to cAMP. We report that even KCTD with relatively weak Gβγ interaction profile in our IP experiments (KCTD4 and TNFAIP1) enabled potent reduction of cAMP signal strength in cultured neurons. Therefore, the physiological roles of diverse KCTD may be significant with regard to GPCR signaling. Recent studies have also demonstrated that elimination of Cullin3 also blocks cyclase sensitization (52, 53), which supports a key role for KCTD scavenging of Gβγ in cAMP signaling in a process independent of modulating Gβγ abundance. Indeed, we also observed that cAMP sensitization was blunted by overexpression of the KCTD17 C terminus alone, which is void of the BTB domain required for Cullin3 recognition.

We utilized the cAMP pathway to examine how principles that guide KCTD–Gβγ interaction impact signal transmission in live cells. This was a rational starting point given significant alterations in cAMP signaling have been linked to KCTD knockout (24, 25) whereas KCTD overexpression influence downstream to cAMP has not yet been reported. Future studies in additional Gβγ-dependent signaling modalities, such as K^+^ flux through GIRK channels, will be of great interest. Previous work has demonstrated the effect of some KCTD in modulating kinetics of current responses from potassium channels in hippocampal neurons (11, 13, 54). Moreover, as Gβγ engage effectors in a spatial manner (6, 7, 8), the potential for KCTD to regulate intracellular Gβγ outcomes may be of interest as well. In addition to the cytosol, KCTD5 has also been localized to the nucleus (55), a compartment where Gβγ dimers are increasingly being recognized to direct signaling (56). Although localization patterns are beginning to emerge for other KCTDs as well (19, 57, 58, 59), the functional significance of such spatial arrangements has yet to be determined.

### Clinical perspective

From a broader perspective, greater insight toward the molecular details guiding KCTD interaction with Gβγ and thus GPCR signaling at large may contribute to better understanding the emerging spectrum of pathophysiology linked to the diverse KCTD family. In addition to a growing list of KCTDs associated with oncogenic hallmarks in cancer (15), a number of KCTD are connected to cognitive disorders and neurodevelopmental disease (14). In particular, movement disorders have arisen from multiple variants in either KCTD7 (60) or KCTD17 (61, 62). Mutations in Gβ1 also cause neurodevelopmental and motor impairments (63), thus linking Gβγ subunits to similar disease states as KCTD. Moreover, *Kctd5*^*+/−*^ mice exhibit significant reductions in motor coordination (25) whereas both *Kctd12* and *Kctd16* knockout mice phenocopy elements of neuropsychiatric disorders (64, 65). Thus, preclinical models are likely to continue advancing our understanding of KCTD in health and disease. Our findings here shed light on how interaction with Gβγ may be involved in such processes.

## Experimental procedures

### Animal subjects

All experimental procedures utilizing mice were approved by Augusta University’s Institutional Animal Care and Use Committee in compliance with guidelines set by the National Institutes of Health. Animals were maintained under standard housing conditions with a 12 h light/dark cycle where all mice had continuous access to food and water. The generation of *Drd1aCre* (Drd1-Cre; EY262; Research Resource Identifier: MMRRC_017264-UCD) and *CAMPER* (C57BL/6-Gt(ROSA)26Sortm1(CAG-ECFP∗/Rapgef3/Venus∗)Kama/J; RRID: IMSR_JAX:032205) mouse lines were previously described (32, 66). Mouse genotypes were verified through standard PCR amplification from genomic DNA samples using primers previously described for each line. Homozygous *CAMPER* mice (*CAMPER*^*+/+*^) were crossed with *Drd1aCre* positive homozygous *CAMPER* (*Drd1aCre*-*CAMPER*^*+/+*^) to obtain pups for primary neuron cultures.

### Bioinformatics

Phylogenetic tree was constructed from human ORF KCTD protein sequences that were aligned in UniProt and rendered from Phylo.io in horizontal format. Amino acid identity matrices were calculated from human KCTD protein sequences utilizing Clustal2.1 Multiple Sequence Alignment. Individual comparison of protein sequences was based on alignments generated from T-COFFEE.

### Molecular cloning

D2R (67), GαoA (complementary DNA Resource Center #GNA0OA0000), Venus 156-239-Gβ1 (27), Venus 1-155-Gγ2 (27), pmCherry-N1 (Takara Bio; catalog no.: 632523), pmVenus-N1 (68), masGRK3ct (69), masGRK3ct-Nluc (29), PTX-S1 (70), ^T^Epac^VV^ (31), AC5 (37), KCTD2-myc (Origene; catalog no.: RC218957), KCTD5-myc (Origene; catalog no.: RC200180), KCTD8-myc (Origene; catalog no.: RC223904), KCTD12-myc (Origene; catalog no.: RC204577), KCTD16-myc (Origene; catalog no.: RC211283), and KCTD17-myc (Origene; catalog no.: RC206070) were previously described. The remaining KCTD ORF were cloned into pCMV6 vector in frame with C-terminal myc-FLAG epitope tags utilizing the In-Fusion Snap Assembly Kit (Takara Bio; catalog no.: 638948). The following human ORFs were codon optimized: KCTD1, KCTD3, KCTD7, KCTD13, KCTD14, KCTD18, KCTD19, SHKBP1, TNFAIP1, BTBD10. The following KCTD ORFs were synthesized from mouse complementary DNA library: KCTD4, KCTD6, KCTD9, KCTD10, KCTD11, KCTD15, KCTD20, KCTD21, and KCNRG. C-terminal Nluc constructs were generated from original pCMV6-KCTD constructs by inserting codon-optimized Nluc (Twist Biosciences) followed by a stop codon at the EcoRV site preceding the myc-FLAG tags. The NotI site was used for KCTD3 and KCTD18, whereas XhoI site was used for BTBD10. KCTD chimeras and mutants were generated by T4 ligation of synthesized DNA (Twist Biosciences) into BamHI/NotI-digested pCMV6. A KpnI site was introduced between synthesized BTB and C-terminal regions for seamless assembly of chimeras. N-terminal mCherry-KCTD constructs were generated by T4 ligation of synthesized DNA (Twist Biosciences) into BsrGI/MfeI-digested pmCherry-N1. C-terminal KCTD-mCherry constructs were generated by T4 ligation of synthesized mCherry ORF DNA (Twist Biosciences) into MluI/XhoI site of KCTD2-myc-FLAG, KCTD5-myc-FLAG, and KCTD17-myc-FLAG.

### Cell culture

Transformed HEK cells (Lenti-X 293T; Takara Bio, catalog no.: 632180) were maintained in a 37 °C humidified incubator with 5% CO_2_ grown in Dulbecco's modified Eagle's medium (Gibco; catalog no.: 11995) supplemented with 10% fetal bovine serum (FBS), minimum Eagle’s medium nonessential amino acids, 100 units/ml penicillin, and 100 μg/ml streptomycin. Cells were transfected with polyethylenimine (Polysciences; #23966-100) in OptiMEM (Gibco; catalog no.: 11058021) on culture vessels or glass coverslips coated with PDL (Gibco; catalog no.: A38904).

### BRET assay

Cells were transfected with D2R, GαoA, Venus 156-239-Gβ1, Venus 1-155-Gγ2, and Nluc-tagged constructs in a 0.5:1:1:1:1 ratio and harvested 18 h later. Cells were washed once with 5 mM EDTA in PBS and then incubated in the same buffer for 10 min to detach. Cells were then centrifuged at 750*g* for 5 min at room temperature and resuspended in PBS containing 0.5 mM MgCl_2_ and 0.1% glucose. Approximately 100,000 cells were transferred per well to a 96-well white opaque plate followed by an equal volume of dopamine (200 μM) or buffer. Following a 5-min room temperature incubation, an equal volume of freshly prepared 2× Nano-Glo Luciferase Assay Substrate (Promega; catalog no.: N1110) was added in the same buffer. End-point BRET measurements were then recorded on a SpectraMax microplate reader (Molecular Devices) utilizing dual PMT for simultaneous detection of Nluc (460 nm) and Venus (530 nm) emission. BRET signal was calculated by dividing acceptor (Venus) by donor (Nluc) wavelength. BRET fold change was calculated by dividing the agonist-induced BRET signal by the basal BRET signal. All experiments were performed at room temperature.

### IP

Approximately 18 to 30 h after transfection, cell pellets were resuspended (PBS supplemented with 150 mM NaCl, 0.5% *n*-dodecanoylsucrose, 30 μM AlF4- [as indicated in text], Roche cOmplete protease inhibitor; catalog no.: 11836170001) and lysed by sonication (15 s; 30% power; FisherBrand; catalog no.: FB50110). The lysates were then centrifuged for 15 min at 12,000 rpm at 4 °C, and the supernatant was split into two tubes: (1) 15% was utilized to probe protein level in the total lysate and (2) 85% was utilized for IP. For the IP, the supernatant was mixed with 10 μl Dynabeads Protein G (Invitrogen; catalog no.: 10003D) and 1 μg anti-GFP antibody (Roche; catalog no.: 11814460001) for 2 h while rotating at 4 °C. Samples were then washed three times for 10 min before elution in LDS sample buffer (National Diagnostics; catalog no.: EC-887) and heating in a 37 °C water bath for 15 min.

### Western blot

Protein concentrations from total lysate supernatants were calculated (Pierce 660 nm protein assay reagent; Thermo Scientific, catalog no.: 22660), and samples were diluted to the same concentration in LDS sample buffer (National Diagnostics; catalog no.: EC-887) and heated in a 37 °C water bath for 15 min. Total lysate (5–10 μg total protein; typically one tenth of the 15% total lysate supernatant fraction) and IP samples (20 μl; 40% of eluent) were subject to SDS polyacrylamide gel electrophoresis, transferred to polyvinylidene difluoride membranes, and incubated with 5% dry nonfat milk (Boston Bioproducts; catalog no.: P-1400) in PBS containing 0.1% Tween-20 (PBST). Membranes were then incubated with primary antibody in 1% milk in PBST as indicated in the text: mouse anti-GFP (Roche; catalog no.: 11814460001), rabbit anti-myc tag (Cell Signaling Technology; catalog no.: 2278), and rabbit anti-mCherry (ProteinTech; catalog no.: 26765-1-AP). Followed by washing in PBST, membranes were incubated with secondary antibodies conjugated to horseradish peroxidase in 1% milk in PBST: mouse anti-rabbit (Jackson ImmunoResearch; catalog no.: 211-032-171) and goat antimouse (Jackson ImmunoResearch; catalog no.: 115-035-174). Protein bands were visualized digitally following application of ECL reagent with the KwikQuant Imager (Kindle Biosciences; catalog no.: D1001).

### Primary culture

Mouse pups (from *Drd1aCre-CAMPER*^*+/+*^ and *CAMPER*^*+/+*^ breeding pairs) were sacrificed at P0 to remove the brain and isolate the striatum, as previously described (25, 32, 71). The striata were dissected in cold Hank’s balanced salt solution (HBSS) supplemented with 20% FBS, 4.2 mM NaHCO_3_, and 1 mM Hepes. Pooled striata were then washed in HBSS (without FBS) and digested for 15 min at 37 °C in a pH 7.2 buffer containing (in millimolar): NaCl (137), KCl (5), Na2HPO4 (7), Hepes (25), and 0.3 mg/ml papain (Worthington). Striata were then washed three times with HBSS (with 20% FBS), three times with HBSS (without FBS), and three times with growth media (Neurobasal-A supplemented with 2 mM GlutaMAX, 2% B27 supplement, 100 units/ml penicillin, and 100 μg/ml streptomycin). Striatal tissue was then mechanically dissociated by multiple passes through a standard P1000 pipette in growth media containing DNAse I (0.05 U/μl). Neurons were then plated on poly-d-lysine-coated German glass coverslips and maintained at 37 °C with 5% CO_2_ in a humidified incubator. At DIV2 and every 3 days thereafter, half of the culture media was replaced with fresh growth media. Cultures were transfected with Lipofectamine 2000 at DIV5 with pmCherry-N1 and the indicated KCTD-myc-FLAG construct.

### cAMP imaging

For live imaging, cells grown on glass coverslips were transferred to a custom chamber with constant perfusion (2 ml/min) of a recording buffer that consisted of (in millimolar): NaCl (125), KCl (2.5), CaCl2 (2), MgCl_2_ (2), NaH_2_PO_4_ (1.25), NaHCO_3_ (25), glucose (25), and Hepes (5). Experiments were performed through a 20× objective lens mounted on a custom Olympus fluorescence microscope. The FRET donor (mTurquoise) was excited by white light illumination (CoolLED pE-300lite) filtered to 436 nm (Chroma ET436/20×). FRET donor (mTurquoise; 455–500 nm) and acceptor (mVenus; 500–600 nm) images were simultaneously captured by splitting emission (Cairn OptoSplit II; Chroma ET480/40m and T455lp) for acquisition through a Hamamatsu sCMOS (ORCA-Flash 4.0). Images were captured at 10 s intervals, and ImageJ was used to calculate FRET from total cellular intensity, as previously described (25, 32, 33, 72). Isoproterenol and dopamine were bath applied in the recording buffer as indicated in the text. All experiments were performed at room temperature.

### Statistical analysis

At least three biological replicates were performed for each experiment. Bar graphs represent mean ± SEM overlaid with plots of each individual data point. Statistical analysis was performed with GraphPad Prism 9 (GraphPad Software, Inc). One-way ANOVA compared with control sample with Dunnett test was utilized for experimental comparisons with utilization of symbols to indicate statistical significance as indicated in the appropriate figure legends. A family wise alpha threshold and confidence level was set at 0.05 (95% confidence interval).

## Data availability

The main text and supporting information contain all associated data, methods, and material sources.

## Supporting information

This article contains supporting information.

## Conflict of interest

The authors declare that they have no conflicts of interest with the contents of this article.
