# Supplementary figures and images for: Multiple potassium channel tetramerization domain (KCTD) family members interact with Gβγ, with effects on cAMP signaling
Source: J Biol Chem. 2023 Feb 1;299(3):102924. doi: 10.1016/j.jbc.2023.102924 (PMC9976452; doi:10.1016/j.jbc.2023.102924)

**A**

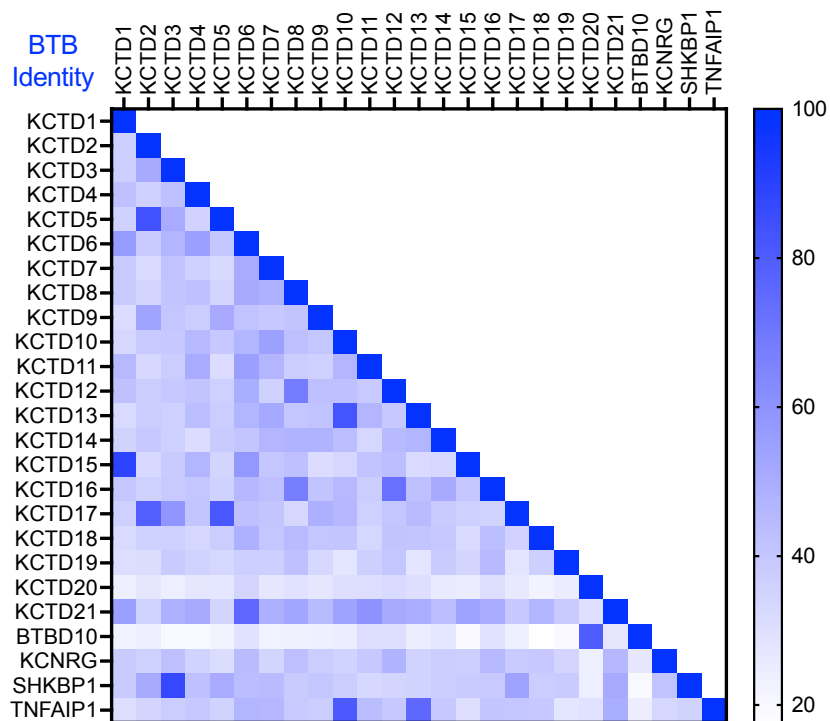

# B

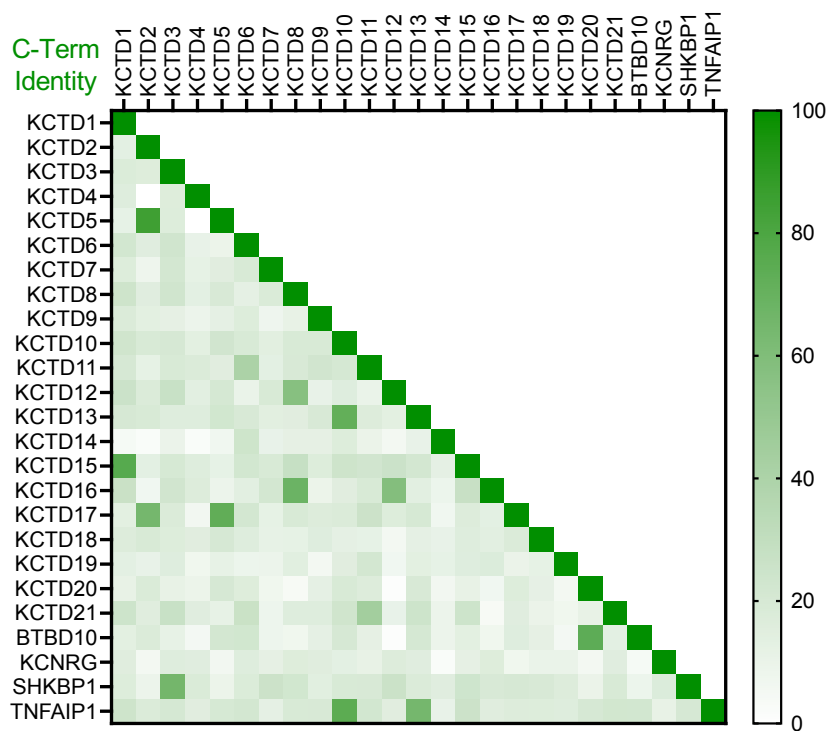

Supplement: Supplemental figure 1 [file mmc1.pdf]
